# A Novel Antimicrobial Peptide Derived from Bony Fish IFN1 Exerts Potent Antimicrobial and Anti-Inflammatory Activity in Mammals

**DOI:** 10.1128/spectrum.02013-21

**Published:** 2022-03-15

**Authors:** Xun Xiao, Hao Lu, Wentao Zhu, Yanqi Zhang, Xingchen Huo, Chunrong Yang, Shaobo Xiao, Yongan Zhang, Jianguo Su

**Affiliations:** a Department of Aquatic Animal Medicine, College of Fisheries, Huazhong Agricultural University, Wuhan, China; b Laboratory for Marine Biology and Biotechnology, Pilot National Laboratory for Marine Science and Technology, Qingdao, China; c State Key Laboratory of Agricultural Microbiology, College of Veterinary Medicine, Huazhong Agricultural University, Wuhan, China; d Hubei Hongshan Laboratory, Engineering Research Center of Green development for Conventional Aquatic Biological Industry in the Yangtze River Economic Belt, Ministry of Education, Wuhan, China; Lerner Research Institute

**Keywords:** gcIFN-20, antimicrobial peptide, bactericidal activity, lipopolysaccharide neutralization, anti-inflammation

## Abstract

Type I interferons (IFN-Is) are critical antiviral cytokine in innate immunity but with limited direct defense ability against bacterial infections in mammals. In bony fish, despite all the IFN-Is (IFN1-4) act in antiviral immunity, studies demonstrate that IFN1 can remarkably contribute to host defense against bacterial infections. In this study, we found that IFN1 from grass carp (*Ctenopharyngodon idella*) contains an unusual cationic and amphipathic α-helical region (named as gcIFN-20, sequence: SYEKKINRHFKILKKNLKKK). The synthesized peptide gcIFN-20 could form α-helical structure in a membrane environment and exerts potent antimicrobial activity against multiple species of Gram-negative (G^−^) and Gram-positive (G^+^) bacteria with negligible toxicity. Mechanism studies showed gcIFN-20 kills G^+^ bacteria through membrane disruption and cytoplasm outflow while G^−^ bacteria through membrane permeation and protein synthesis inhibition. In two mouse bacterial infection models, gcIFN-20 therapy could significantly reduce tissue bacterial loads and mortalities. In addition to the direct antibacterial activity, we also found that gcIFN-20 could significantly suppress the lipopolysaccharide (LPS)-induced pro-inflammatory cytokines *in vitro* and *in vivo*, obviously alleviated lung lesions in a mouse endotoxemia model. The mechanism is that gcIFN-20 interacts with LPS, causes LPS aggregation and neutralization. The antimicrobial and anti-inflammatory activities *in vivo* of gcIFN-20 in mammalian models suggested a promising agent for developing peptide-based antibacterial therapy.

**IMPORTANCE** Type I interferons play crucial role in antiviral immunity in both vertebrates and invertebrates. The powerful antimicrobial activity is recently reported in nonmammalian vertebrates. The present study identified a novel antimicrobial peptide (gcIFN-20) derived from grass carp interferon 1, found gcIFN-20 exhibits forceful bactericidal and anti-inflammatory activity in mammals, and efficient therapeutic effect against two clinical severe extraintestinal pathogenic Escherichia coli and a mouse endotoxemia models. The antimicrobial mechanisms are membrane disruption and cytoplasm overflow for Gram-positive bacteria, while membrane permeation and protein synthesis inhibition for Gram-negative bacteria. The anti-inflammatory mechanisms can be aggregating and neutralizing lipopolysaccharide to attenuate the binding with receptors and facilitate phagocytosis. The results indicate that gcIFN-20 can be a promising novel therapeutic agent for bacterial diseases and inflammatory disorders, especially as a potential weapon for multidrug resistant strain infections.

## INTRODUCTION

Host defense peptides (HDPs) are ancient potent weapons against bacterial pathogens, which are essential components of immune system in all the vertebrates (1). Generally, most natural HDPs vary in length (usually 12 to 50 aa) and are characterized by a high proportion of hydrophobic residues and a net positive charge (typically +2 to +9) (2). Despite these universal features, there is high sequence and structural diversity among HDPs from different species. The structures of HDPs ranges from α-helical and/or β-sheet-containing structures or linear peptides (1, 2). With the change of environment, HDPs can form different conformations and mediate the change of biological activity (3). In addition to promoting direct microbial killing, HDPs can affect the host response to infection in multiple ways, including the modulation of chemokine and cytokine production, angiogenesis, endotoxin neutralization, and wound healing (1). The chemical space of HDPs includes all the peptide sequences of a given length with particular amino acid residues and their location within the sequence that can promote or inhibit particular activities (4). Thus, each biological function of HDPs can be envisioned as a series of overlapping activity landscapes that can be optimized independently (4). Due to their diverse antimicrobial and immunomodulatory properties, HDPs and their derivatives, natural defense modulators, are promising candidates for novel anti-infective agents (5).

Type I interferons (IFN-Is) are a class of cytokines initially induced by viral or bacterial infections (6). The secreted IFN-Is could bind to IFN-I receptors, which are widely expressed in a variety of tissues and cells and induces the production of a variety of interferon-stimulated genes to limit viral infections (7, 8). The innate immunity largely depend on production of IFN-Is to provide the first line of defense against viral infections. So far, at least 20 (13 IFN-α subtypes, IFN-β, IFN-ω, IFN-ε, IFN-κ, IFN-δ, IFN-ζ, and IFN-τ) and 4 (IFN1, IFN2, IFN3 and IFN4) IFN-Is have been identified in mammals and bony fishes, respectively (9–11). Although some studies indicated that many bacterial pathogens could also induce the production of IFN-Is (12, 13), mammalian IFN-Is exerted very limited resistance to bacterial and mainly play an important role in antiviral infections (14, 15). Due to highly similar crystal structure (16) and antiviral function (17) among IFN-Is between mammals and fishes, piscine IFN-Is were ordinarily thought to be mainly involved in the treatment of viral infections similar with mammalian IFN-Is.

It is worth noting that zebrafish (Danio rerio) IFNφ1 can protect the fish from bacterial infection besides viral infection (18). We recently discovered that grass carp (*Ctenopharyngodon idella*) IFN1 (highly homologous to zebrafish IFNφ1) exhibits potent direct antimicrobial activity *in vitro* and *in vivo* (19). Hence, bony fish IFN1 may represent a highly effective antimicrobial effector, which has selectively evolved under the pressure of complex bacterial and viral pathogens in water. Therefore, it can be an excellent molecule for studying fish-specific HDPs. Although the existing research implicates the antibacterial defense of fish IFN1, further functions and mechanisms remain largely unknown.

## RESULTS

### IFN1 contains a cationic, amphipathic novel α-helical peptide (gcIFN-20).

**FIG 1:**
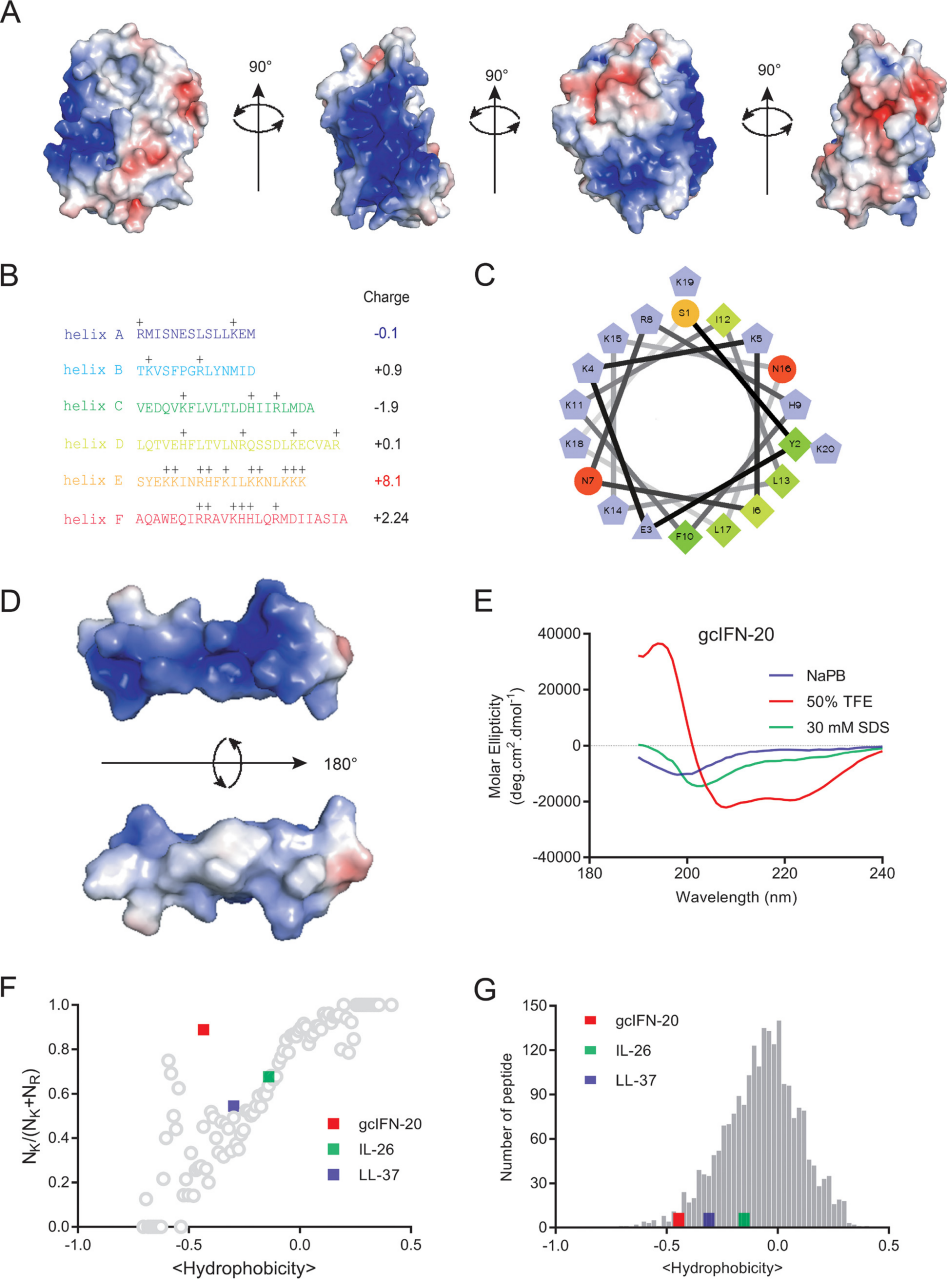
The structural features of gcIFN-20. (A) Color-coded electrostatic potentials were mapped onto the surfaces of gcIFN1. Areas with positive charges are shown in blue, negative charges are in red, and hydrophobic residues are in white. (B) Amino acid sequences and positive charges in gcIFN1 α-helices. Total net charge of every helix is shown on the right. (C) Helical wheel plot of gcIFN-20 illustrates the facial amphipathicity along the helical axis. Charged hydrophilic residues are in violet (positive residues K, R, H in pentagons and negative residue E in triangle), uncharged hydrophilic residues are in orange (S) and red (N), and hydrophobic residues are in green (F, L, I, Y). (D) Color-coded electrostatic potentials were mapped onto the surface of gcIFN-20. Areas with positive charges are shown in blue, negative charge is in red, and hydrophobic residues are in white. (E) The CD spectra of gcIFN-20 (70 μM) in 50 μM NaPB, 50% TFE, or 30 mM SDS. (F) Relationship between positively charged amino acid residues (N_K_/_[NK + NR]_) and average peptide hydrophobicity for 2,237 cationic AMPs in the AMP database (gray circles). Human LL-37 and IL-26 were plotted for references. (G) Comparison of hydrophobicity between gcIFN-20 and AMPs. Histograms depict the distribution of hydrophobicity among the 2,237 cationic AMPs in the AMP database (gray bars). Human LL-37 and IL-26 were presented for references.

Our previous study indicated that gcIFN1 (grass carp IFN1) possesses unusual cationicity and powerful bactericidal activity in comparison with gcIFN2-4 (19). To gain further insight into the cationicity of gcIFN1, we analyzed the distribution of electrostatic charge on the surface of gcIFN1, and the most cationic charges (blue color) of gcIFN1 concentrate a patch (Fig. 1A), which contains eight lysine (40%, a lysine-rich region), one arginine and one histidine residues (45% alkaline amino acid, 50% positively charged amino acid) (Fig. 1B). In contrast, the other α-helixes of gcIFN1 demonstrate low net charges and alkaline amino acids (Fig. 1B). Helical wheel and surface-charge distribution analyses of helix E indicate that the cationic charges mainly distribute on one side of the helical axis, on the opposite side of this cationic cluster, a hydrophobic patch is present (Fig. 1C and D). The predominant polar amino acids and the hydrophobic residues distribute on the opposite side, which indicates that helix E is a cationic and amphipathic peptide. Since helix E is composed of 20 amino acids, we named it as gcIFN-20 (sequence: SYEKKINRHFKILKKNLKKK). Furthermore, the circular dichroism (CD) spectra of gcIFN-20 in different solutions were measured to verify the accuracy of the specific cationic and amphiphilic α-helical structure. As shown in Fig. 1E, gcIFN-20 maintains 100% random coil structure in water or saline solution but forms 100% helical structure in 50% TFE solution, which simulates a hydrophobic membrane environment. Thus, gcIFN-20 is a highly cationic and amphipathic peptide and forms complete α-helical structure in membrane simulation environment, representing a typical structure similar with nature antimicrobial peptides (AMPs).

To date, more than 3,200 AMPs have been listed in the antimicrobial peptide database 3 (https://aps.unmc.edu/). According to the previously observed trend (20), we further analyzed the amino acid compositions in gcIFN-20 between NK/(NK + NR) (the ratio of the number of lysines/total number of lysines and arginines) and average peptide hydrophobicity based on the Eisenberg consensus scale of 2,237 cationic AMPs in database (21). To our surprise, the position of gcIFN-20 seriously deviates from the 2,237 cationic AMP trendline (Fig. 1F). Meanwhile, the hydrophobicity of gcIFN-20 is within the range of hydrophobicity of a relatively small fraction of AMPs in independent evaluation of the hydrophobic residue content (Fig. 1G). Human LL-37 and IL-26, two typical cationic amphiphilic AMPs, were employed as references. Taken together, gcIFN-20 may be a rare AMP which possesses lower hydrophobicity and higher lysine content than other known AMPs.

### GcIFN-20 possesses direct bactericidal activity and negligible toxicity.

**FIG 2:**
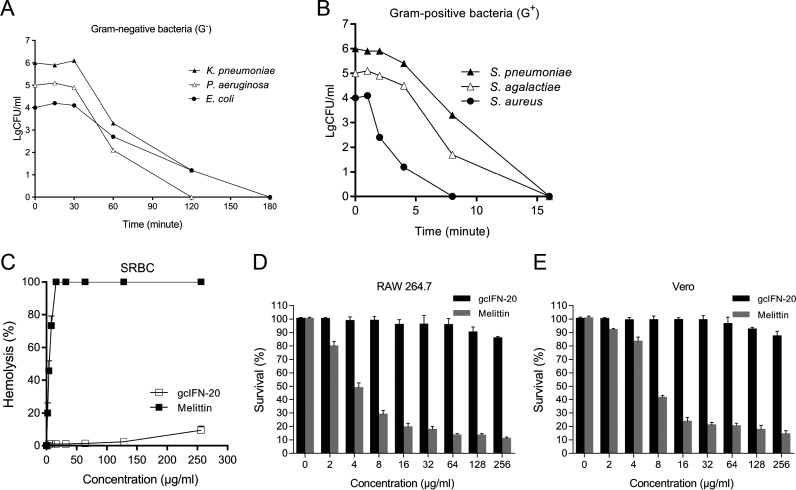
Potent bactericidal activity and low toxicity of gcIFN-20 *in vitro* and *in vivo*. (A and B) Time-kill curves of gcIFN-20 (4×MBC_90_) against G^−^ and G^+^ bacteria, respectively. The six bacterial strains are K. pneumoniae (ATCC 13883), P. aeruginosa (ATCC 9027), E. coli (ATCC 25922), S. pneumoniae (ATCC 49619), S. agalactiae (ATCC 13813) and S. aureus (ATCC 25923). (C) Hemolytic activity of gcIFN-20 was measured with SRBC, and melittin was used as the control. (D and E) The cytotoxicity of gcIFN-20 toward RAW 264.7 cells and Vero cells were examined by MTT assay, respectively. All the experiments were performed in triplicate. Data were presented as means ± SD.

GcIFN-20 has structural features similar with natural AMPs (22, 23), we wondered whether gcIFN-20 has bactericidal property. Our results indicated gcIFN-20 has broad-spectrum robust bactericidal activity against Gram-negative (G^−^) bacteria (Klebsiella pneumoniae, Pseudomonas aeruginosa, and Escherichia coli) (Fig. 2A) and Gram-positive (G^+^) bacteria (Streptococcus pneumoniae, Streptococcus agalactiae, and Staphylococcus aureus) (Fig. 2B). The MBC_90_ is 8–16 μg/mL for G^−^ bacteria and 2–4 μg/mL for G^+^ bacteria, respectively (Table 1). Besides the common laboratory pathogenic strains, gcIFN-20 also demonstrates potent bactericidal activity against clinical MDR G^+^ and G^−^ bacteria (Table 2). After that, we evaluated the toxicity of gcIFN-20 against mammalian cells. Compared with the melittin (control), gcIFN-20 displays lower hemolytic activity toward sheep red blood cell (SRBC) (Fig. 2C), and weakly inhibits cell proliferation of RAW 264.7 cells (murine monocyte/macrophage cell line) (Fig. 2D) and Vero cells (African green monkey kidney cell line) (Fig. 2E) even at high concentration of 256 μg/mL (about 100 μM), which indicated that gcIFN-20 has weak toxicity (IC_50_>100 μM) toward mammalian cells. Together, these results indicated that gcIFN-20 possessed efficiently antimicrobial ability and negligible toxicity.

**TABLE 1:** GcIFN-20 antimicrobial activity against laboratory strains

Bacterial strain	MIC (μg/mL)	MBC (μg/mL)	MBC_90_ (μg/mL)	G^+^/G^−^^*a*^
Klebsiella pneumoniae (ATCC 13883)	32	64	8	G^−^
Pseudomonas aeruginosa (ATCC 9027)	32	32	16	G^−^
Escherichia coli (ATCC 25922)	32	64	16	G^−^
Streptococcus pneumoniae (ATCC 49619)	8	16	2	G^+^
Streptococcus agalactiae (ATCC 13813)	8	8	2	G^+^
Staphylococcus aureus (ATCC 25923)	16	16	4	G^+^

a G^+^/G^−^ indicates Gram-positive and Gram-negative bacteria, respectively.

**TABLE 2:** gcIFN-20 antimicrobial activity against MDR bacteria

MDR bacterial strain	MIC (μg/mL)	MBC (μg/mL)	MBC_90_ (μg/mL)	G^+^/G^−^
Escherichia coli (ATCC 35218)^*a*^	32	64	16	G^−^
Escherichia coli (1103)	32	32	8	G^−^
Escherichia coli (1205)	32	32	8	G^−^
Escherichia coli (1306)	32	64	16	G^−^
Escherichia coli (PCN033)^*b*^	32	64	16	G^−^
Escherichia coli (RS218)^*b*^	32	32	8	G^−^
Salmonella enterica (S17)	32	32	8	G^−^
Klebsiella pneumoniae (K13)	32	64	16	G^−^
Staphylococcus aureus (ATCC 43300)^*a*^	16	16	4	G^+^
Streptococcus suis (1504)	8	16	2	G^+^

a Indicates typical drug resistant strain, not MDR.

b Indicates clinical ExPEC. RS218 is not MDR.

### GcIFN-20 disrupts the membrane integrity of S. aureus and inhibits protein synthesis of E. coli.

**FIG 3:**
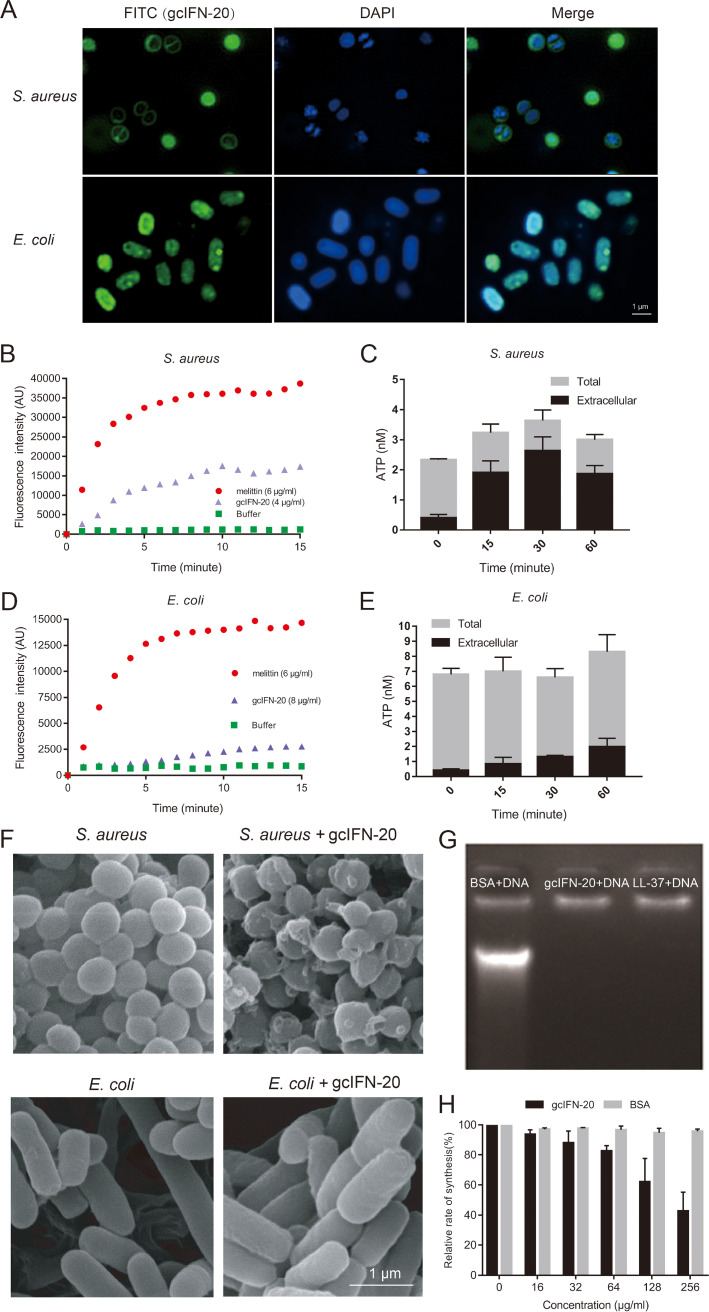
Bactericidal mechanisms of gcIFN-20. (A) Localizations of FITC-gcIFN-20 in bacteria. S. aureus and E. coli were incubated with FITC-gcIFN-20 (0.5×MBC_90_), respectively. The cultures were washed and stained with DAPI (blue). Images were taken using SIM. (B) SYTOX Green uptake in S. aureus. Melittin was used as the positive control. Fluorescence was recorded every minute. (C) The measurement of total and extracellular ATP at different time after treatment of S. aureus with gcIFN-20 (2×MBC_90_) (*n* = 3). (D) SYTOX Green uptake in E. coli. (E) The measurement of total and extracellular ATP at different time after treatment of E. coli with gcIFN-20 (2×MBC_90_) (*n* = 3). (F) S. aureus (upper panel) and E. coli (lower panel) were cultured for 15 min without or with 10 μM gcIFN-20 and then visualized by SEM, respectively. (G) Gel shift assay for E. coli DNA mixed with gcIFN-20. BSA and LL-37 serve as the negative and positive controls, respectively. (H) The effect of gcIFN-20 and BSA (control) with different concentrations on the relative rate of E. coli bacterial protein synthesis in a cell-free assay. All the experiments were run in triplicate. Data were shown as means ± SD.

The bactericidal mechanisms of cationic AMPs are commonly through the disruption of cell membrane or targeting some intracellular components such as DNA (24). To investigate the possible mechanism for microbial-killing function of gcIFN-20, we employed the FITC-labeled gc-IFN20 to visualize the location of gcIFN-20 in bacteria through structured illumination microscopy (SIM). As shown in Fig. 3A, gcIFN-20 can effectively adsorb on the surface of S. aureus and E. coli and penetrate into the cytoplasm of E. coli. Then, we employed SYTOX Green uptake assay and ATP leakage assay to evaluate the effect of gcIFN-20 on the bacterial membrane integrity. Both results support that gcIFN-20 could rapidly disrupt bacterial membranes of S. aureus (Fig. 3B and C). However, a significant difference is that gcIFN-20 does not obviously destroy the integrity of E. coli membrane (Fig. 3D and E). Furthermore, scanning electron microscopy (SEM) results also support this finding. S. aureus shows noticeable membrane pore formation and cytoplasm outflow at 15 min after gcIFN-20 treatment, but E. coli still maintains the regular rod-like structure post gcIFN-20 treatment (Fig. 3F). We wondered whether gcIFN-20 kills E. coli by targeting the intracellular molecular without destroying the outer membrane integrity. We found that gcIFN-20 can bind directly to the genomic DNA of E. coli, similar with LL-37 (positive control) (Fig. 3G). Furthermore, gcIFN-20 but not BSA (negative control) can inhibit the protein synthesis in E. coli cell-free expression system in a concentration dependent manner (Fig. 3H). Together, our data indicated gcIFN-20 kills bacteria by disrupting the membrane integrity and cytoplasm overflow for G^+^ bacteria while penetrating the cytomembrane, binding to DNA and inhibiting protein synthesis for G^−^ bacteria.

### GcIFN-20 demonstrates forceful antimicrobial activity against ExPEC in mice.

**FIG 4:**
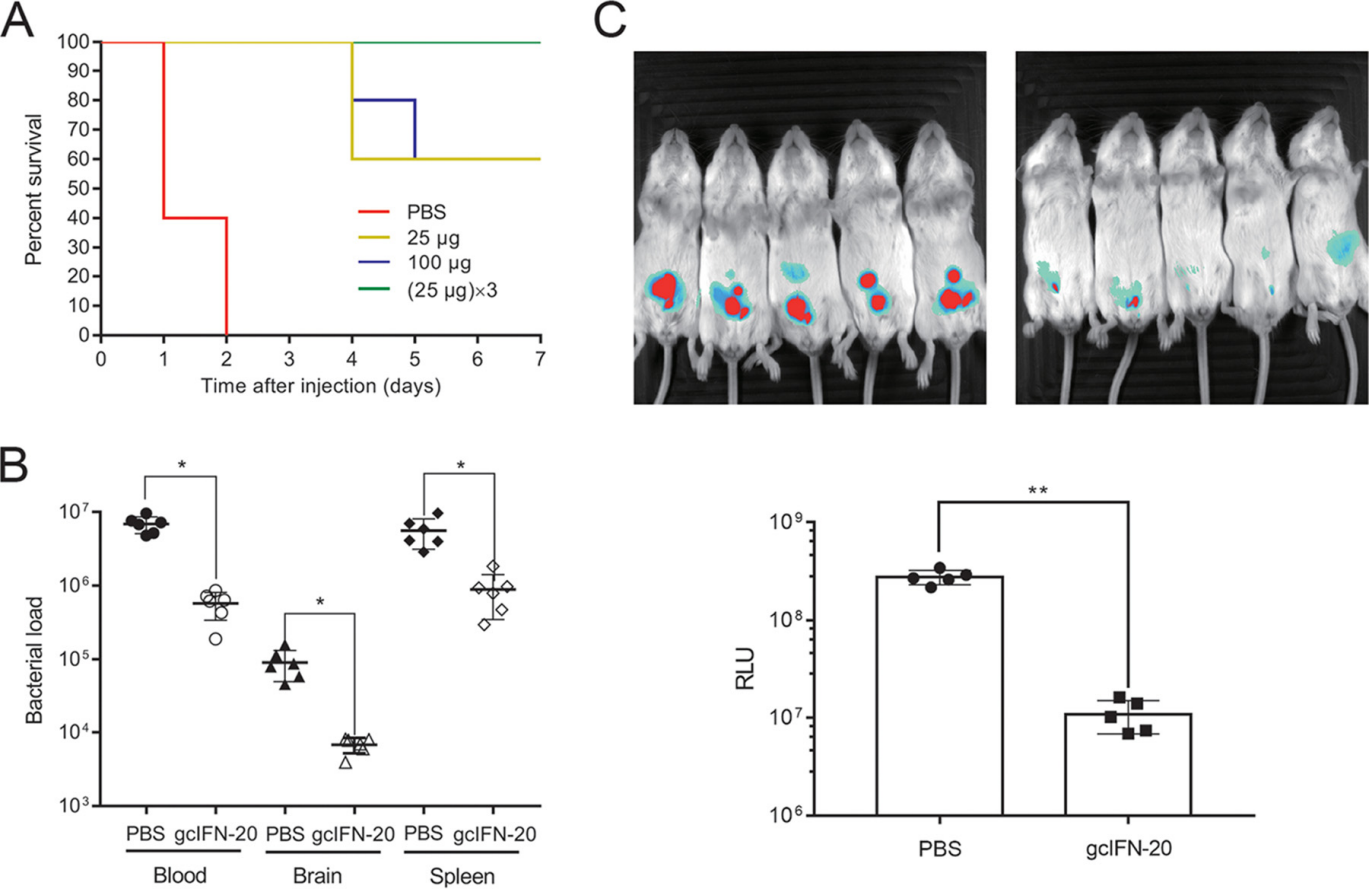
Potent bactericidal activity of gcIFN-20 *in vivo*. (A) Survival rates in mice were examined by injection with E. coli PCN033 and gcIFN-20 (*n* = 10). (B) Bacterial loads in mouse blood (CFU/mL), brain (CFU/g), and spleen (CFU/g) (gcIFN-20 25 μg/mouse) at 12 h post bacterial injection (*n* = 6). (C) IVIS analysis of the antimicrobial activity of gcIFN-20 (25 μg/mouse) *in vivo* against E. coli RS218. Bacterial loads were displayed in the image with an overlay of bioluminescence. False color imaging for strong luminescence was in red and mild luminescence in blue. The total flux was quantified by the IVIS software (*n* = 5). Data were presented as means ± SD. *In vivo* data were statistically analyzed via unpaired nonparametric Mann-Whitney U-test. * indicates *P* ≤ 0.01.

We wondered whether gcIFN-20 has antibacterial activity *in vivo*? Using mouse infection model with a clinical severe ExPEC strain PCN033 (25), we found that gcIFN-20 with different doses can completely protect all the mice from death on day 3. Although mice given a single dose of gcIFN-20 only have a 60% survival rate on day 7, mice given three doses of gcIFN-20 on the first day still maintain a 100% survival rate on day 7 (Fig. 4A). Furthermore, we examined the effect of gcIFN-20 on the tissue bacterial loads. As shown in Fig. 4B, the tissue bacterial titers have about 10-fold reduction in blood, brain, and spleen at 12 h post bacterial injection. Moreover, we established a bioluminescent ExPEC strain RS218 to visually examine the antimicrobial activity by *in vivo* imaging system (IVIS). The decrease of bacterial loads *in vivo* was confirmed by reduced bioluminescence in mice. As shown in Fig. 4C, the RLU values very significantly weakened in gcIFN-20 treatment group at 4 h post bacterial injection. Together, these data indicated that gc-IFN20 possesses efficiently antimicrobial ability *in vivo*.

### GcIFN-20 exerts potent lipopolysaccharide (LPS)-neutralization activity by promoting LPS aggregation.

**FIG 5:**
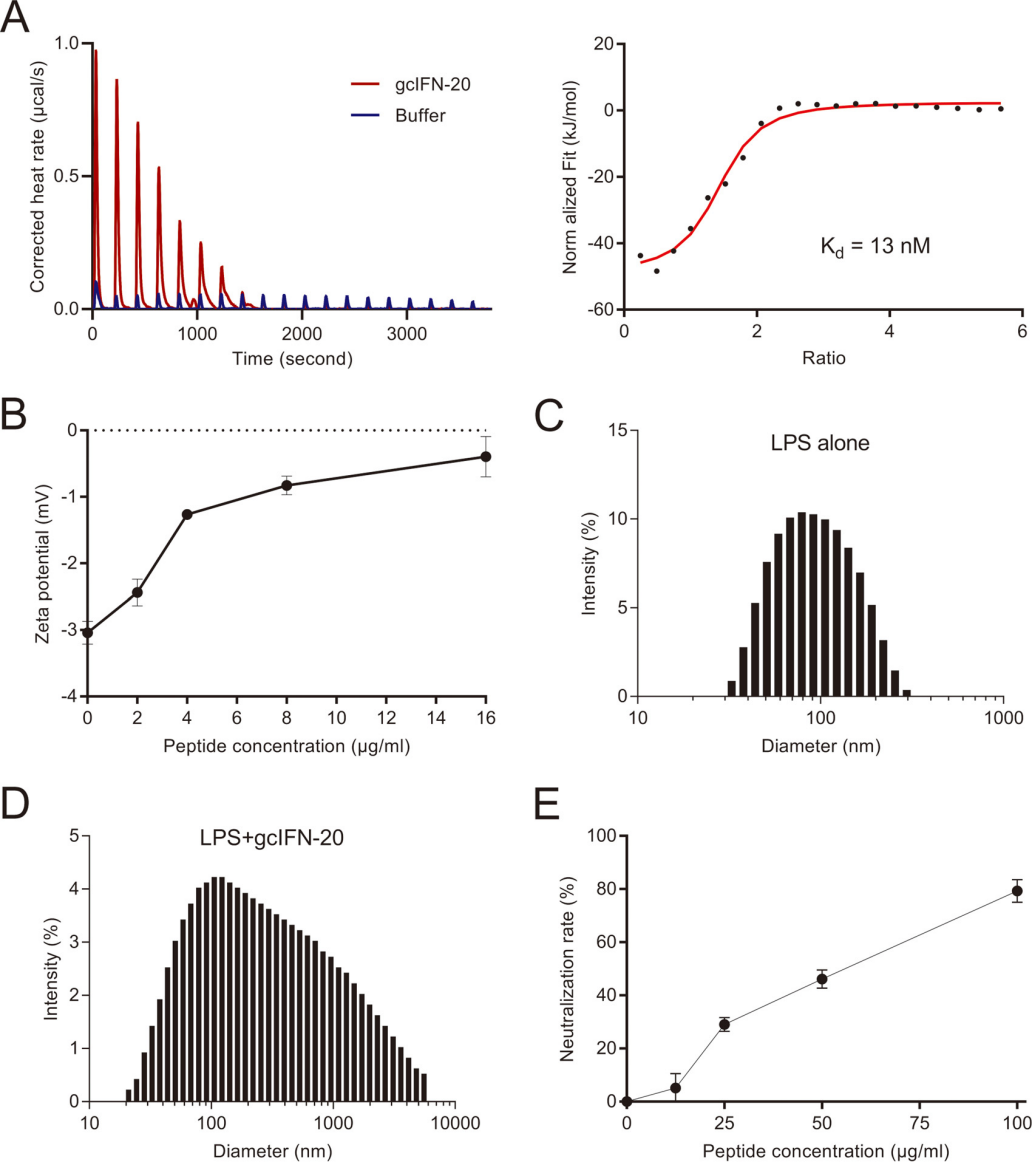
LPS neutralization activity and mechanisms of gcIFN-20. (A) The specific interaction between gcIFN-20 and LPS was investigated by ITC. The corrected titration data and integrated heat measurements were shown on the left and right plots, respectively. The solid line on the right panel represents the best fit to a one-site binding model for the interaction between gcIFN-20 and LPS. (B) The effect of gcIFN-20 on the zeta potentials of LPS aggregates. (C) Particle size distribution of ultrapure LPS. (D) Particle size distribution of LPS in the presence of gcIFN-20 (200 μg/mL). (E) LPS neutralization activity of gcIFN-20 was examined by LAL assay. All the experiments were conducted in triplicate. Data were shown as means ± SD.

LPS released from the killed bacteria can cause endotoxemia, we wondered whether gcIFN-20 can neutralize LPS. First, we investigated the interaction between LPS and gcIFN-20 by isothermal titration calorimetry (ITC) and zeta potential assay. The ITC result reveals a strong interaction between gcIFN-20 and LPS (K_d_ = 13 nM) (Fig. 5A), and the zeta potential measurements are consistent with the ITC result (Fig. 5B). The negative charges on LPS were effectively neutralized when we mixed LPS with increasing concentrations of gcIFN-20 (Fig. 5B). The aggregation state of LPS is closely related to its pro-inflammatory activity. Further, we examined the particle size and distribution of by dynamic light scattering (DLS) assay. Ultrapure LPS has a size distribution center at 102.6 nm (Fig. 5C). However, the large particles with size distribution center at 615.0 nm form through the extensive aggregation caused by gcIFN-20 (Fig. 5D), suggesting that gcIFN-20 can aggregate LPS to form larger polymer. Finally, we confirmed that gcIFN-20 prevents LPS activity by LAL assay. As shown in Fig. 5E, gcIFN-20 neutralizes more than 80% LPS at a concentration of 100 μg/mL. Together, these results indicated that gcIFN-20 exerts potent LPS-neutralization activity by promoting LPS aggregation in addition to direct antimicrobial ability.

### GcIFN-20 remarkably suppresses the LPS-stimulated inflammation *in vitro* and *in vivo*.

**FIG 6:**
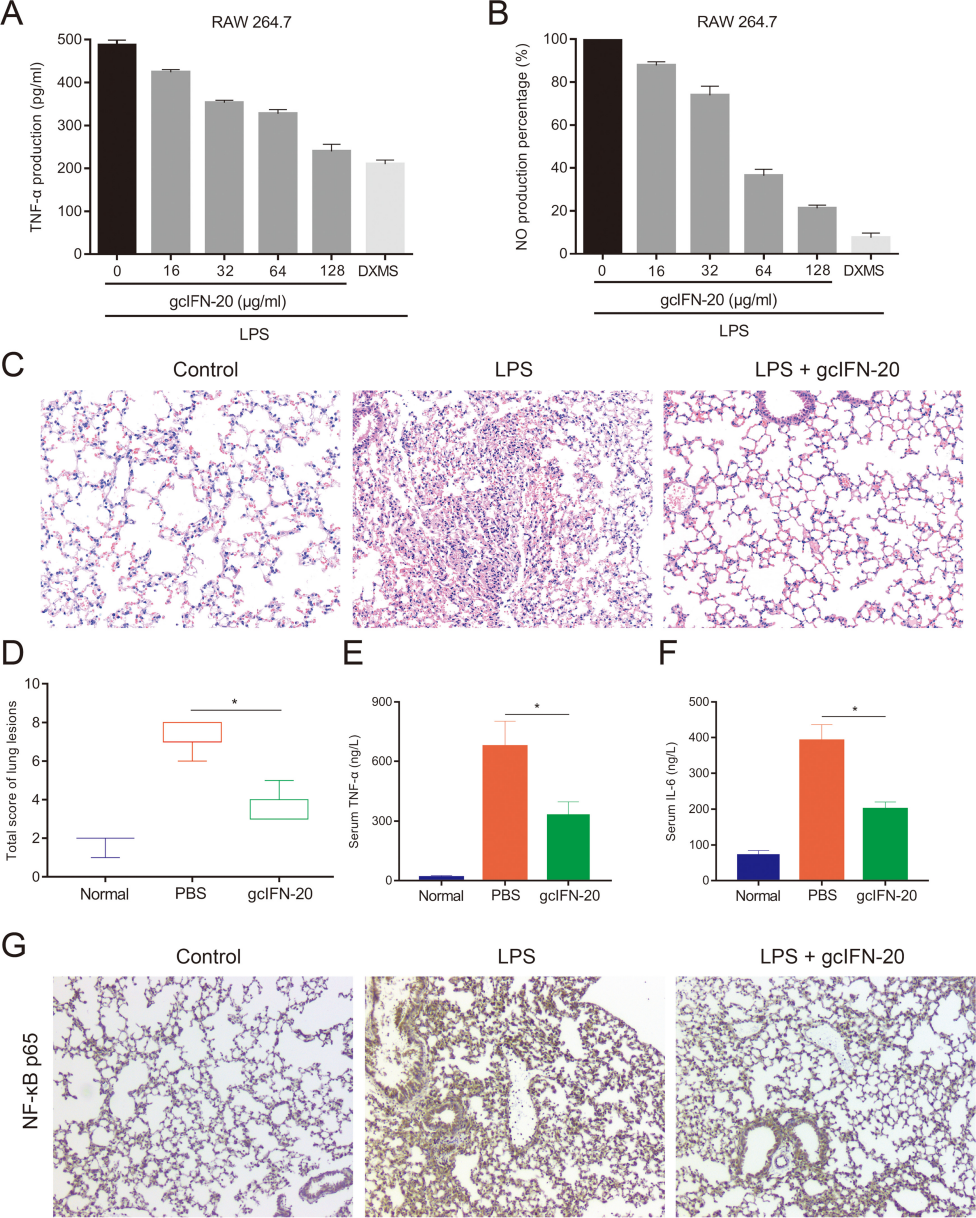
Anti-inflammatory activity of gcIFN-20 in LPS-induced RAW 264.7 cells and mice. The productions of TNF-α (A) and NO (B) were investigated in RAW 264.7 cells, respectively. Dexamethasone serves as the control. (C) The protective effect of gcIFN-20 on lung tissue in an endotoxemia mouse model (*n* = 6). (D) The total lung lesion scores were generated in a blinded manner by a certified pathologist according to a semiquantitative scoring method. (E and F) The suppression of the LPS-stimulated TNF-α and IL-6 release in endotoxemia mice by gcIFN-20, respectively. (G) Immunohistochemical analysis of NF-κB p65 in the lung slices of experimental mice. All the experiments were performed in triplicate. Data were presented as means ± SD.

LPS can cause serious harmful inflammatory responses (26). Tumor necrosis factor-α (TNF-α) and nitric oxide (NO) represent the major inflammatory products released by cells following exposure to LPS. After confirming that gcIFN-20 can neutralize LPS as above, we further assessed the effects of gcIFN-20 on LPS-stimulated TNF-α and NO content in RAW 264.7 cells. We treated the cells with 150 ng/mL LPS in the presence of gcIFN-20 at 0–128 μg/mL. GcIFN-20 remarkably inhibits the productions of TNF-α (Fig. 6A) and NO (Fig. 6B) in a dose-dependent manner.

Furthermore, we investigated the anti-inflammatory effect *in vivo*. In a mouse model of LPS-induced endotoxemia, the lung tissue slices stained with hematoxylin-eosin (HE) were photomicrographed, shown, and scored in Fig. 6C and D, respectively. The LPS group show obvious histological changes characterized by alveolar interstitial congestion, edema, and substantial inflammatory cell infiltration. However, gcIFN-20 group display distinctly reduced lung injury. In addition, the levels of serum pro-inflammatory cytokines TNF-α and interleukin-6 (IL-6) are significantly reduced in gcIFN-20 treated mice (Fig. 6E and F). Nuclear factor-κB (NF-κB) is of central importance in inflammatory responses. The NF-κB p65 subunit immunohistochemistry of mouse lung tissue slice shows evident reduction in the gcIFN-20 group (Fig. 6G). These results indicated that gcIFN-20 can suppress the LPS-induced inflammatory responses *in vitro* and *in vivo*.

## DISCUSSION

IFN-Is are the core component of innate immunity against viral infections. The highly conserved antiviral function has been demonstrated in almost all the vertebrates in which IFN-Is have been found (27, 28). However, our previous study indicated that bony fish IFN1 could effectively kill bacteria through membrane disruption mechanism (19), which means IFN1 associates with the natural HDPs. In this study, we analyzed the sequence composition of gcIFN1 and identified a novel AMP (gcIFN-20) derived from the fifth helical region in gcIFN1.

Our previous research showed that the main difference between IFN1 and the other IFN-Is (IFN2-4) is that IFN1 has high positive charges (19). However, the strong cationic nature of IFN1 cannot fully explain the outstanding antibacterial activity of IFN1 because some cationic proteins do not have direct antibacterial activity. In this study, we observed that most of the basic amino acids of IFN1 concentrate in the fifth α-helical region and the other helical regions of IFN1 are almost electrically neutral or weakly charged. Therefore, we hypothesized that the antibacterial function of IFN1 largely depends on this region. Our follow-up investigations confirmed that the synthesized gcIFN-20 peptide does exhibit powerful antibacterial activity *in vitro* and in two mouse models. This situation is like that of the chemokine family (29, 30), since the antimicrobial property of many chemokines is closely related to the α-helical region.

Further, we investigated the antibacterial property and mechanism of gcIFN-20. We found that the antibacterial activity of gcIFN-20 is coincident with that of IFN1. Both of them have lower MBC_90_ and faster bactericidal activity against G^+^ bacteria. In addition, they kill G^+^ bacteria through a membrane damage mechanism. However, gcIFN-20 exhibits different bactericidal mechanism against G^−^ bacteria. Typically, gcIFN-20 does not cause obvious damage to E. coli cytomembrane but penetrates into the cytoplasm and effectively inhibits the protein synthesis. Therefore, our data demonstrated gcIFN-20 has unique antibacterial mechanisms to kill G^−^ bacteria.

Although a high number of AMPs have strong bactericidal effect *in vitro*, most of them lack effective antibacterial activity *in vivo* due to the complexity of animal internal environment (31). In this study, we confirmed that gcIFN-20 not only exerts potent antimicrobial ability *in vitro*, but also has an efficient therapeutic effect on two clinical isolates of ExPEC (PCN033 and RS218) *in vivo*. In addition, some amphiphilic α-helical peptides may have highly toxic to eukaryotic cells (32, 33) and are not conducive to clinical drug development. Although gcIFN-20 can also form a complete α-helix conformation in a membrane-simulated environment, but we did not observe obvious cytotoxicity to eukaryotic cells. PN5, a novel AMP from pine needles, has an α-helical amphipathic structure and shows no detectable hemolytic activity or cytotoxicity at the antimicrobial concentrations, thus, they can serve as an alternative therapeutic agent to be used in the food industry (34). AMPs are considered a possible solution to conquer the MDR problem at postantibiotics era (35–37), our data suggested gcIFN-20 may be a potential anti-infective clinical agent.

During G^−^ bacterial infections, LPS plays a crucial role in the pathophysiology of the inflammatory sepsis and shock (38, 39). Another noticeable finding of this study is that gcIFN-20 demonstrates remarkable anti-inflammatory activity in the LPS-induced acute lung injury model and in *in vitro* experiments. Similarly, a synthetic AMP GW-A2 inhibits inflammatory response and NLRP3 inflammasome by neutralizing LPS and ATP, which provides a foundation for the design of rational therapeutics for inflammation-related diseases (40). The anti-inflammatory mechanisms of gcIFN-20 depend on LPS aggregation and neutralization, which coincide with modified Bip-P-113 (41) and polymyxin B (42). GcIFN-20 aggregates and covers LPS by electrostatic attraction, which facilitates macrophage phagocytosis and degradation, and blocks LPS interaction with its cell receptors for inflammatory responses. The statement that LPS is covered by gcIFN-20 was verified by LAL assay. Recently, a designed α-helical hybrid AMP PA-13 shows broad-spectrum antibacterial activity by membrane depolarization and permeabilization, and anti-inflammatory activity via LPS neutralization (43). However, the experiments *in vivo* are absent. Another novel LPS-binding AMP esculentin-1 GN (GLFSKKGGKGGKSWIKGVFKGIKGIGKEVGGDVIRTGIEIAACKIKGEC) from frog skin with antimicrobial and anti-inflammatory activities *in vitro* and *in vivo* can be an excellent template for designing new antibiotic formulations (44). However, it is much bigger. The efficient anti-inflammatory effect of gcIFN-20 *in vitro* and *in vivo* further confers strong therapeutic potential in G^−^ bacterial infections.

In summary, the present study identified a novel cationic amphiphilic α-helical peptide gcIFN-20 derived from gcIFN1, which possesses broad-spectrum forceful bactericidal activity, anti-inflammatory activity and neglectable toxicity to mammals *in vitro* and *in vivo*. The functional mechanism of gcIFN-20 involves in the first step of electrostatic attraction toward LPS or PGN on bacterial cell walls; then, forms α-helical structure and disrupts membrane (G^+^) or permeates membrane (G^−^); further, membrane disruption mainly results in cytoplasm outflow and death, while membrane permeation mainly causes gcIFN-20 to bind bacterial DNA to prevent DNA replication, mRNA transcription and protein synthesis, and death; furthermore, the released LPS from killed bacteria (G^−^) is aggregated and neutralized by gcIFN-20 for anti-inflammation. The potent antimicrobial and anti-inflammatory activities *in vivo* endow gcIFN-20 with promising clinical application prospect in mammals in bacterial infections, especially in MDR bacteria (superbugs) infections.

## MATERIALS AND METHODS

### Ethics statement.

BALB/c mice were obtained from the Laboratory Animal Center of Huazhong Agricultural University (Wuhan, China). All the experiments were performed in accordance with the guidelines of the Laboratory Animal Center of Huazhong Agricultural University. The protocols were approved by the Ethical Committee on Animal Research at Huazhong Agricultural University (HZAUMO-2017-038). All the efforts were made to minimize animal suffering.

### Bacterial isolates.

E. coli (ATCC 25922) and (ATCC 35218), P. aeruginosa (ATCC 9027), K. pneumoniae (ATCC 13883), S. aureus (ATCC 25923), (ATCC 43300), S. agalactiae (ATCC 13813), and S. pneumoniae (ATCC 49619) were obtained from American Type Culture Collection (ATCC). Clinical isolates of E. coli (1103), (1205), (1306), Salmonella enterica (S17), K. pneumoniae (K13), Streptococcus suis (1504), and extraintestinal pathogenic E. coli (ExPEC) PCN033 were isolated from clinical specimens of swine origin and were identified by Key Laboratory of Preventive Veterinary Medicine in Hubei Province (Wuhan, Hubei, China). E. coli (1103), E. coli (1205), E. coli (1306), S. enterica (S17), K. pneumoniae (K13), and S. suis (1504) belong to multidrug resistant (MDR) strains, which are resistant to more than three antibiotics, including ampicillin, cefotaxime, levofloxacin, chloramphenicol, tetracycline, polymyxin B, and sulfamethoxazole-trimethoprim. Susceptibility to antibiotics was tested in accordance with the guidelines of the Clinical & Laboratory Standards Institute.

### Peptides.

LL-37 (sequence: LLGDFFRKSKEKIGKEFKRIVQRIKDFLRNLVPRTES), melittin (sequence: GIGAVLKVLTTGLPALISWIKRKRQQ), gcIFN-20 were synthesized in GenScript Biotech Corporation (NJ, USA).

### Protein modeling and charge analysis.

Protein modeling was based on the SWISS-MODEL Template Library (https://www.swissmodel.expasy.org/). The predicted protein structures were downloaded as PDB files and then imported into PyMOL (PyMOL Molecular Graphics System, Version 1.3, 2011; Schrödinger) to generate the protein models. The structural homology of gcIFN1 was carried out by PyMOL. Further net charge distribution was performed by APBS software plug-in (https://www.easycounter.com/report/poissonboltzmann.org). The online software (http://www.novopro.cn/tools/protein_iep.html) was used for net charge analyses of gcIFN1 helixes and the online software (https://heliquest.ipmc.cnrs.fr/cgi-bin/ComputParams.py) was employed for the helical wheel analysis.

### CD spectroscopy.

The CD spectra were measured by a Jasco J-1500 spectropolarimeter (Jasco, Japan). The measurements were performed with a peptide concentration of 70 μM in a 10 mm quartz cuvette at 37°C in triplicate. The peptide secondary structures in 50 μM sodium phosphate buffer (NaPB), 50% trifluoroethanol (TFE; vol/vol), or 30 mM SDS were monitored in the range of 190–240 nm. To account for instrumental difference, background value (detected at 250 nm, where no peptide signal presents) was subtracted.

### Antimicrobial assay.

Antimicrobial activity is evaluated by CFU assay (45). The bacteria were cultured overnight in LB medium at 37°C and then subcultured to achieve mid-logarithmic phase growth. The bacteria were collected, washed with PBS, and then diluted to a final concentration of 10^5^ CFU/mL in an incubation buffer (10 mM Tris-HCl, 5 mM glucose, pH 7.4). A bacterial suspension (100 μL) was incubated with 100 μL of peptide for 3 h at 37°C. After that, the suspension was spread on LB plates for 12–16 h. The number of colonies were counted by two independent investigators. The MBC was determined as the minimum concentration that killed all the bacteria after 3 h of incubation. The MBC_90_ of gcIFN-20 was defined as the minimum concentration that killed >90% of the CFU of bacteria after 3 h of incubation. The MIC of gcIFN-20 peptide was defined as the minimum concentration of the peptide that the bacteria could not grow in the 96-well plate in 3 h.

### Hemolytic activity of gcIFN-20 to SRBC.

Hemolytic activity was assessed using the SRBC pellets according to the previous method (23). In brief, SRBC suspension (Final erythrocyte concentration of 5 × 10^7^ cells/mL) was mixed with the different concentrations of gcIFN-20 (8, 16, 32, 64, 128, and 256 μg/mL, 100 μL) or melittin and incubated for 4 h at 37°C. After that, centrifugation was performed at 800 *g* for 10 min to collect the cells for the measurement of absorbance at 540 nm. For calibration, 100% hemolysis was achieved by incubating the samples with 1% Triton X-100, whereas the negative control was incubated with PBS only. Each measurement was performed in triplicate.

### Cytotoxicity of gcIFN-20 to mammalian cells.

Cytotoxicity was measured in RAW 264.7 and Vero cells by 3-(4,5-Dimethylthiazol-2-yl)-2,5-Diphenyltetrazolium Bromide (MTT) assay. The cells were seeded in a 96-well plate at 10^4^ cells/well, and serial dilutions of gcIFN-20 or melittin were added into each well. PBS was used as the negative control. After incubation for 1 h, we replaced the medium with fresh medium containing 10% MTT solution (Promega) for 4 h at 37°C under 5% CO_2_. The absorbance at 595 nm was measured by a microplate reader of multiwavelength measurement system. Each measurement was performed in triplicate. In both hemolytic activity and cytotoxicity assays, IC50 (half maximal inhibitory concentration) is defined as the minimum concentration of peptides that causes half of the cell lysis or death.

### SIM assay.

The localizations of gcIFN-20 in bacteria were monitored by SIM (46). E. coli or S. aureus were cultured at 37°C overnight in LB medium and then subcultured to achieve mid-logarithmic phase growth. 100 μL of bacteria (5 × 10^7^ CFU/mL) were incubated with FITC-labeled gcIFN-20 at a concentration of 0.5 × MBC_90_ for 1 h at 37°C. The bacteria were stained with DAPI (Invitrogen) for 15 min. After that, the cells were washed three times with PBS. The localizations of FITC-labeled gcIFN-20 were observed by SIM (Nihonika, Japan).

### Membrane integrity assay.

The S. aureus (ATCC 25923) and E. coli (ATCC 25922) cells were employed to detect the membrane integrity with SYTOX Green (Invitrogen) by the method described previously (46). In brief, S. aureus or E. coli bacterial suspensions were grown in LB medium to exponential phase. Bacterial suspensions (5 × 10^7^ CFU/mL) were incubated with gcIFN-20 at indicated concentrations with shaking at 37°C, collecting and washing cells every minute during the 15-minute experimental period. SYTOX Green (1 μM) was incubated with the treated samples for 15 min in the dark. The fluorescence intensity was measured at wavelengths 485 nm and 520 nm filters for excitation and emission. Melittin and BSA were employed as positive and negative controls, respectively.

### ATP leakage assay.

The total and extracellular ATP concentration of S. aureus (ATCC 25923) and E. coli (ATCC 25922) was determined as described previously (47), by using an ATP bioluminescence kit (Invitrogen). The amount of ATP in the samples was calculated using an ATP standard curve. Each measurement was performed in triplicate.

### SEM assay.

E. coli or S. aureus was cultured at 37°C overnight in LB medium and then subcultured to achieve mid-logarithmic phase growth. A total of 400 μL of E. coli or S. aureus (5 × 10^7^ CFU/mL) were centrifuged at 4000 g for 10 min. The cells were washed three times with PBS and incubated with 10 μM gcIFN-20 or buffer (20 mM Tris-HCl, 10 mM NaCl, pH 7.4) at 37°C for 15 min. Then, the cells were fixed with 2.5% glutaraldehyde at 4°C overnight. The cells were dehydrated by 10% to 100% ethanol series (10 min per step) and coated with gold. Finally, the cells were examined by a scanning electron microscope (JSM-840) (48).

### Gel retardation assay.

The binding ability of gcIFN-20 to bacterial DNA was tested by gel retardation assay (46). In brief, gcIFN-20, LL-37 (positive control), and BSA (negative control) were incubated with bacterial DNA (final concentrations: 3 μg/mL DNA and 1 μM gcIFN-20, LL-37 or BSA) for 15 min at 37°C, respectively. After that, the mobility of the mixtures was examined by electrophoresis on 1% agarose gel.

### Cell-free protein synthesis assay.

The E. coli S30 T7 high-yield protein expression system (Promega) were employed to detect the inhibition effect of gcIFN-20 peptide on bacterial protein-synthesis as previous described (49). The hydrolyzation of substrate to yield o-nitrophenyl (ONP) was measured as an increase in absorbance at 420 nm at 37°C in a Multi-Mode Microplate Readers for 1 h at intervals of 15 min. The experimental group contain 150 μg/mL gcIFN-20 peptide and the control group contain 150 μg/mL BSA. All tests were performed in parallel at least three times.

### Compositional comparison between gcIFN-20 and AMPs.

We compared the amino acid compositions of human IL-26 (50), LL-37 (51), gcIFN-20 with known AMPs for generating membrane curvature based on the geometry of membrane destabilization (20). A set of 2,237 cationic AMP sequences were sourced from the AMP database (21). The minimum and maximum <hydrophobicity> values within the set of AMP sequences were defined as the <hydrophobicity> range. This range was divided into 100 equal bins, into which the peptides were partitioned. N_K_/(N_K_ + N_R_) represents the ratio of the lysines to lysines plus arginines. For each bin, N_K_/(N_K_ + N_R_) versus <hydrophobicity> was plotted using MATLAB. To compare the hydrophobicity, the <hydrophobicity> histogram values for the set of AMPs were constructed with 50 bins using MATLAB. The hydrophobicities of IL-26, LL-37, and gcIFN-20 were superimposed over the AMP histogram.

### Mouse acute peritonitis infection model.

ExPEC PCN033 (52, 53) was cultured overnight, harvested, washed, and diluted by PBS. The bacteria (5 × 10^7^ CFU) were intraperitoneally injected into 4-week-old female BALB/c mice (*n* = 10/group). After 1 h, the animals were intraperitoneally injected with 100 μl of 25 μg of gcIFN-20, 100 μg of gcIFN-20 or PBS (negative control). The fourth group was intraperitoneally injected with gcIFN-20 thrice (25 μg each instance at 4 h interval). The mice were monitored every 6 h for 7 days for clinical symptoms of disease and mortality. The bacterial titers in blood, brain, and spleen were determined. Mice were euthanized at 12 h post bacterial injection (*n* = 6/group). The blood, brain and spleen were collected, weighed, homogenated, diluted in PBS, spread, and grown on LB plates for 12–16 h at 37°C. The E. coli colonies were counted by two independent investigators.

### IVIS assay.

Plasmid pBEN276 (54) was employed to construct a stable reporting system within the chromosome of ExPEC RS218 (25) for extended trial *in vivo*. The 5 × 10^8^ CFU bioluminescent RS218 (confirmed by pretest) was intraperitoneally injected into 4-week-old female BALB/c mice (*n* = 5/group). After 1 h, the animals were intraperitoneally injected with 100 μl of 25 μg gcIFN-20 or PBS. At 4 h post bacterial injection, the mice were anesthetized and transferred to the IVIS (Newton 7.0, Vilber Lourmat, France). The luminescence was tested with an exposure time of 5–15 sec. The imaging system translates the data into false color images that depict the area of strong luminescence with red and mild luminescence with blue. The bioluminescence intensity is proportional to the number of bacteria. The total flux of the target area was quantified by the IVIS software.

### ITC assay.

ITC experiment was performed on Waters Nano ITC (55). E. coli O111:B4 LPS (Sigma-Aldrich) was vortexed for 15 min. A typical titration involved 20 injections of 1.0 mM gcIFN-20 (2 μL per injection, except the first injection of 3 μL) into the microcalorimetric cell containing 50 mM LPS at 180 sec intervals. The reaction was stirred continuously at 250 rpm at 37°C. Raw data were corrected for the heat of gcIFN-20 dilution in buffer and integrated using Microcal origin 5.0 supplied by the manufacturer. One-site binding model was used to fit the data by nonlinear least square method.

### Zeta potential.

E. coli O111:B4 LPS was vortexed for 15 min and fixed at 1 mg/mL. The electrophoretic mobilities of LPS were determined in the absence and presence of gcIFN-20 at different concentrations (0–16 μM) by phase analysis light scattering (PALS) using disposable zeta cells as previously described (Zetasizer Nano ZS) (41). Zeta potential was calculated by the mobility of LPS.

### DLS assay.

To investigate the particle size and distribution, DLS experiments were performed (Zetasizer Nano ZS). E. coli O111:B4 LPS (1 mg/mL) was incubated with different concentrations (0–10 μM) of gcIFN-20. Three measurements with 13 runs each were performed at 37°C. We examined the size of gcIFN-20 in buffer, and we did not observe any gcIFN-20 aggregate. The particle size of LPS in buffer was tested as control. The size distribution of LPS aggregates in 10 μM gcIFN-20 was shown.

### LPS neutralization.

To examine the endotoxin neutralization capacity of gcIFN-20, we used the quantitative chromogenic limulus amebocyte lysate (LAL) kit (Xiamen Bioendo Technology Co., Ltd.). GcIFN-20 was diluted in pyrogen-free water provided with the kit (final concentrations: 100, 50, 25, 12.5, and 0 μg/mL gcIFN-20), incubated with 1 unit of LPS at 37°C for 30 min, added an equal volume of LAL reagents for 10 min, then added 100 μL of chromogenic substrate for 15 min, finally, 50 μL of 25% acetic acid was added to terminate the reaction. The absorbances at 405/545 nm were measured by the microplate reader of a multiwavelength measurement system. Each measurement was performed in triplicate.

### Inhibition of LPS-induced inflammation *in vitro*.

RAW 264.7 cells were cultured in 24-well plates with 3 × 10^5^ cells per well for 24 h at 37°C under 5% CO_2_. The cells were washed three times with PBS, and 300 μL of Dulbecco's Modified Eagle Medium (DMEM) containing LPS (E. coli O26:B6 LPS [Sigma-Aldrich], 150 ng/mL) and gcIFN-20 (0–128 μg/mL) was added to each well. Dexamethasone (1 μM) serves as the positive control. After incubation overnight, the supernatant was harvested by centrifuging the culture at 1500 *g* for 10 min at 4°C. The concentration of NO was determined by a nitric oxide assay kit (Beyotime Biotechnology), while the concentration of TNF-α was examined by mouse ELISA kits (Multisciences).

### Mouse endotoxemia model.

The endotoxemia model was employed as previously reported (41, 56). Briefly, E. coli O26:B6 LPS (Sigma-Aldrich) was administered intranasally to induce endotoxemia (18 mg/kg). PBS and 5 mg/kg gcIFN-20 were intraperitoneally injected at 1 h after LPS treatment, respectively. Blood was collected via the tail vein at 6 h post LPS treatment. Whole blood was centrifuged at 3000 rpm at 4°C for 10 min, and the supernatant was collected. Serum TNF-α and IL-6 were measured using mouse ELISA kits (Multisciences). At 12 h after LPS treatment, all the mice were sacrificed, and lung tissues were collected. The pathological changes in lungs were evaluated after the slices were stained with HE, and NF-κB p65 protein was analyzed by immunohistochemistry.

### Statistical analysis.

Statistical analyses were performed using the two-tailed unpaired Student's *t* test in *in vitro* experiments and the two-tailed unpaired nonparametric Mann-Whitney U-test in *in vivo* experiments. *, *P* ≤ 0.05; **, *P* ≤ 0.01.

### Data availability.

The data that support the findings of this study are available on request from the corresponding author.

## References

[1] Hancock RE, Haney EF, Gill EE. 2016. The immunology of host defence peptides: beyond antimicrobial activity. Nat Rev Immunol 16:321–334. doi:10.1038/nri.2016.29. 27087664

[2] Nguyen LT, Haney EF, Vogel HJ. 2011. The expanding scope of antimicrobial peptide structures and their modes of action. Trends Biotechnol 29:464–472. doi:10.1016/j.tibtech.2011.05.001. 21680034

[3] Hancock REW, Alford MA, Haney EF. 2021. Antibiofilm activity of host defence peptides: complexity provides opportunities. Nat Rev Microbiol 19:786–797. doi:10.1038/s41579-021-00585-w. 34183822

[4] Haney EF, Straus SK, Hancock REW. 2019. Reassessing the host defense peptide landscape. Front Chem 7:43. doi:10.3389/fchem.2019.00043. 30778385 PMC6369191

[5] Magana M, Pushpanathan M, Santos AL, Leanse L, Fernandez M, Ioannidis A, Giulianotti MA, Apidianakis Y, Bradfute S, Ferguson AL, Cherkasov A, Seleem MN, Pinilla C, de la Fuente-Nunez C, Lazaridis T, Dai T, Houghten RA, Hancock REW, Tegos GP. 2020. The value of antimicrobial peptides in the age of resistance. Lancet Infect Dis 20:e216–e230. doi:10.1016/S1473-3099(20)30327-3. 32653070

[6] McNab F, Mayer-Barber K, Sher A, Wack A, O'Garra A. 2015. Type I interferons in infectious disease. Nat Rev Immunol 15:87–103. doi:10.1038/nri3787. 25614319 PMC7162685

[7] Coccia EM, Battistini A. 2015. Early IFN type I response: learning from microbial evasion strategies. Semin Immunol 27:85–101. doi:10.1016/j.smim.2015.03.005. 25869307 PMC7129383

[8] Levraud JP, Jouneau L, Briolat V, Laghi V, Boudinot P. 2019. IFN-stimulated genes in zebrafish and humans define an ancient arsenal of antiviral immunity. J Immunol 203:3361–3373. doi:10.4049/jimmunol.1900804. 31732531

[9] Hardy MP, Owczarek CM, Jermiin LS, Ejdebäck M, Hertzog PJ. 2004. Characterization of the type I interferon locus and identification of novel genes. Genomics 84:331–345. doi:10.1016/j.ygeno.2004.03.003. 15233997

[10] Liu F, Bols NC, Pham PH, Secombes CJ, Zou J. 2019. Evolution of IFN subgroups in bony fish-1: group I-III IFN exist in early ray-finned fish, with group II IFN subgroups present in the holostean spotted gar, *Lepisosteus oculatus*. Fish Shellfish Immunol 95:163–170. doi:10.1016/j.fsi.2019.10.032. 31626921

[11] Liao Z, Wan Q, Su J. 2016. Bioinformatics analysis of organizational and expressional characterizations of the IFNs, IRFs and CRFBs in grass carp *Ctenopharyngodon idella*. Dev Comp Immunol 61:97–106. doi:10.1016/j.dci.2016.03.020. 27012995

[12] Peignier A, Planet PJ, Parker D. 2020. Differential induction of type I and III interferons by *Staphylococcus aureus*. Infect Immun 88:e00352-20. doi:10.1128/IAI.00352-20. 32690637 PMC7504949

[13] Gutierrez-Merino J, Isla B, Combes T, Martinez-Estrada F, Maluquer De Motes C. 2020. Beneficial bacteria activate type-I interferon production via the intracellular cytosolic sensors STING and MAVS. Gut Microbes 11:771–788. doi:10.1080/19490976.2019.1707015. 31941397 PMC7524384

[14] Peignier A, Parker D. 2021. Impact of type I interferons on susceptibility to bacterial pathogens. Trends Microbiol 29:823–835. doi:10.1016/j.tim.2021.01.007. 33546974 PMC8326292

[15] Parker D, Prince A. 2011. Type I interferon response to extracellular bacteria in the airway epithelium. Trends Immunol 32:582–588. doi:10.1016/j.it.2011.09.003. 21996313 PMC3221817

[16] Hamming OJ, Lutfalla G, Levraud JP, Hartmann R. 2011. Crystal structure of zebrafish interferons I and II reveals conservation of type I interferon structure in vertebrates. J Virol 85:8181–8187. doi:10.1128/JVI.00521-11. 21653665 PMC3147990

[17] Robertsen B. 2006. The interferon system of teleost fish. Fish Shellfish Immunol 20:172–191. doi:10.1016/j.fsi.2005.01.010. 15939626

[18] López-Muñoz A, Roca FJ, Meseguer J, Mulero V. 2009. Newinsights into the evolution of IFNs: zebrafish group II IFNs induce a rapid and transient expression of IFN-dependent genes and display powerful antiviral activities. J Immunol 182:3440–3449. doi:10.4049/jimmunol.0802528. 19265122

[19] Xiao X, Zhu W, Zhang Y, Liao Z, Wu C, Yang C, Zhang Y, Xiao S, Su J. 2021. Broad-spectrum robust direct bactericidal activity of fish IFNφ1 reveals an antimicrobial peptide-like function for type I IFNs in vertebrates. J Immunol 206:1337–1347. doi:10.4049/jimmunol.2000680. 33568398

[20] Schmidt NW, Mishra A, Lai GH, Davis M, Sanders LK, Tran D, Garcia A, Tai KP, McCray PB, Ouellette AJ, Selsted ME, Wong GC. 2011. Criterion for amino acid composition of defensins and antimicrobial peptides based on geometry of membrane destabilization. J Am Chem Soc 133:6720–6727. doi:10.1021/ja200079a. 21473577 PMC3090259

[21] Wang G, Li X, Wang Z. 2016. APD3: the antimicrobial peptide database as a tool for research and education. Nucleic Acids Res 44:D1087–93. doi:10.1093/nar/gkv1278. 26602694 PMC4702905

[22] Jiang M, Yang X, Wu H, Huang Y, Dou J, Zhou C, Ma L. 2020. An active domain HF-18 derived from hagfish intestinal peptide effectively inhibited drug-resistant bacteria *in vitro/vivo*. Biochem Pharmacol 172:113746. doi:10.1016/j.bcp.2019.113746. 31812678

[23] Ma L, Wang Y, Wang M, Tian Y, Kang W, Liu H, Wang H, Dou J, Zhou C. 2016. Effective antimicrobial activity of Cbf-14, derived from a cathelin-like domain, against penicillin-resistant bacteria. Biomaterials 87:32–45. doi:10.1016/j.biomaterials.2016.02.011. 26897538

[24] Zasloff M. 2019. Antimicrobial peptides of multicellular organisms: my perspective. Adv Exp Med Biol 1117:3–6. doi:10.1007/978-981-13-3588-4_1. 30980349

[25] Bloch CA, Rode CK. 1996. Pathogenicity island evaluation in *Escherichia coli* K1 by crossing with laboratory strain K-12. Infect Immun 64:3218–3223. doi:10.1128/iai.64.8.3218-3223.1996. 8757856 PMC174210

[26] Nau R, Eiffert H. 2002. Modulation of release of proinflammatory bacterial compounds by antibacterials: potential impact on course of inflammation and outcome in sepsis and meningitis. Clin Microbiol Rev 15:95–110. doi:10.1128/CMR.15.1.95-110.2002. 11781269 PMC118062

[27] Mesev EV, LeDesma RA, Ploss A. 2019. Decoding type I and III interferon signalling during viral infection. Nat Microbiol 4:914–924. doi:10.1038/s41564-019-0421-x. 30936491 PMC6554024

[28] Gan Z, Chen Shan N, Huang B, Zou J, Nie P. 2020. Fish type I and type II interferons: composition, receptor usage, production and function. Rev Aquacult 12:773–804. doi:10.1111/raq.12349.

[29] Liu B, Wilson E. 2010. The antimicrobial activity of CCL28 is dependent on C-terminal positively-charged amino acids. Eur J Immunol 40:186–196. doi:10.1002/eji.200939819. 19830739 PMC2866449

[30] Margulieux KR, Fox JW, Nakamoto RK, Hughes MA. 2016. CXCL10 acts as a bifunctional antimicrobial molecule against *Bacillus anthracis*. mBio 7:e00334-16. doi:10.1128/mBio.00334-16. 27165799 PMC4959661

[31] Barreto-Santamaría A, Rivera ZJ, García JE, Curtidor H, Patarroyo ME, Patarroyo MA, Arévalo-Pinzón G. 2020. Shorter antibacterial peptide having high selectivity for *E. coli* membranes and low potential for inducing resistance. Microorganisms 8:867. doi:10.3390/microorganisms8060867. 32521823 PMC7356157

[32] Ciornei CD, Egesten A, Bodelsson M. 2003. Effects of human cathelicidin antimicrobial peptide LL-37 on lipopolysaccharide-induced nitric oxide release from rat aorta *in vitro*. Acta Anaesthesiol Scand 47:213–220. doi:10.1034/j.1399-6576.2003.00045.x. 12631052

[33] Gutsmann T, Hagge SO, Larrick JW, Seydel U, Wiese A. 2001. Interaction of CAP18-derived peptides with membranes made from endotoxins or phospholipids. Biophys J 80:2935–2945. doi:10.1016/S0006-3495(01)76259-5. 11371466 PMC1301477

[34] Lee J, Kang HK, Cheong H, Park Y. 2021. A novel antimicrobial peptides from pine needles of *Pinus densiflora* Sieb. et Zucc. against foodborne bacteria. Front Microbiol 12:662462. doi:10.3389/fmicb.2021.662462. 34093476 PMC8172577

[35] Liu Y, Ding S, Shen J, Zhu K. 2019. Nonribosomal antibacterial peptides that target multidrug-resistant bacteria. Nat Prod Rep 36:573–592. doi:10.1039/c8np00031j. 30324212

[36] Khan SN, Khan AU. 2016. Breaking the spell: combating multidrug resistant 'superbugs'. Front Microbiol 7:174. doi:10.3389/fmicb.2016.00174. 26925046 PMC4757689

[37] Mookherjee N, Anderson MA, Haagsman HP, Davidson DJ. 2020. Antimicrobial host defence peptides: functions and clinical potential. Nat Rev Drug Discov 19:311–332. doi:10.1038/s41573-019-0058-8. 32107480

[38] Callejas JL, Moreno E, Martin P, Lopez-Perez L, Ortego N. 2007. Stress-induced pulmonary systolic hypertension in patients with scleroderma. Chest 131:1267. doi:10.1378/chest.06-2430. 17426243

[39] Voss OH, Murakami Y, Pena MY, Lee HN, Tian L, Margulies DH, Street JM, Yuen PS, Qi CF, Krzewski K, Coligan JE. 2016. Lipopolysaccharide-induced CD300b receptor binding to toll-like receptor 4 alters signaling to drive cytokine responses that enhance septic shock. Immunity 44:1365–1378. doi:10.1016/j.immuni.2016.05.005. 27261276 PMC4917413

[40] Li LH, Ju TC, Hsieh CY, Dong WC, Chen WT, Hua KF, Chen WJ. 2017. A synthetic cationic antimicrobial peptide inhibits inflammatory response and the NLRP3 inflammasome by neutralizing LPS and ATP. PLoS One 12:e0182057. doi:10.1371/journal.pone.0182057. 28750089 PMC5531531

[41] Chih YH, Wang SY, Yip BS, Cheng KT, Hsu SY, Wu CL, Yu HY, Cheng JW. 2019. Dependence on size and shape of non-nature amino acids in the enhancement of lipopolysaccharide (LPS) neutralizing activities of antimicrobial peptides. J Colloid Interface Sci 533:492–502. doi:10.1016/j.jcis.2018.08.042. 30176540

[42] Domingues MM, Inácio RG, Raimundo JM, Martins M, Castanho MA, Santos NC. 2012. Biophysical characterization of polymyxin B interaction with LPS aggregates and membrane model systems. Biopolymers 98:338–344. doi:10.1002/bip.22095. 23193598

[43] Klubthawee N, Adisakwattana P, Hanpithakpong W, Somsri S, Aunpad R. 2020. A novel, rationally designed, hybrid antimicrobial peptide, inspired by cathelicidin and aurein, exhibits membrane-active mechanisms against *Pseudomonas aeruginosa*. Sci Rep 10:9117. doi:10.1038/s41598-020-65688-5. 32499514 PMC7272617

[44] Zeng B, Chai J, Deng Z, Ye T, Chen W, Li D, Chen X, Chen M, Xu X. 2018. Functional characterization of a novel lipopolysaccharide-binding antimicrobial and anti-inflammatory peptide *in vitro* and *in vivo*. J Med Chem 61:10709–10723. doi:10.1021/acs.jmedchem.8b01358. 30427189

[45] Dai C, Basilico P, Cremona TP, Collins P, Moser B, Benarafa C, Wolf M. 2015. CXCL14 displays antimicrobial activity against respiratory tract bacteria and contributes to clearance of *Streptococcus pneumoniae* pulmonary infection. J Immunol 194:5980–5989. doi:10.4049/jimmunol.1402634. 25964486

[46] Chen HL, Su PY, Chang YS, Wu SY, Liao YD, Yu HM, Lauderdale TL, Chang K, Shih C. 2013. Identification of a novel antimicrobial peptide from human hepatitis B virus core protein arginine-rich domain (ARD). PLoS Pathog 9:e1003425. doi:10.1371/journal.ppat.1003425. 23785287 PMC3681751

[47] Yasir M, Dutta D, Kumar N, Willcox MDP. 2020. Interaction of the surface bound antimicrobial peptides melimine and Mel4 with *Staphylococcus aureus*. Biofouling 36:1–30. doi:10.1080/08927014.2020.1843638. 33161763

[48] Zhang Y, Xiao X, Hu Y, Liao Z, Zhu W, Jiang R, Yang C, Zhang Y, Su J. 2021. CXCL20a, a teleost-specific chemokine that orchestrates direct bactericidal, chemotactic, and phagocytosis-killing-promoting functions, contributes to clearance of bacterial infections. J Immunol 207:1911–1925. doi:10.4049/jimmunol.2100300. 34462313

[49] Liu Y, Luan C, Xia X, An S, Wang Y. 2011. Antibacterial activity, cytotoxicity and mechanisms of action of cathelicidin peptides against enteric pathogens in weaning piglets. Int J Pept Res Ther 17:175–184. doi:10.1007/s10989-011-9255-y.

[50] Meller S, Di Domizio J, Voo KS, Friedrich HC, Chamilos G, Ganguly D, Conrad C, Gregorio J, Le Roy D, Roger T, Ladbury JE, Homey B, Watowich S, Modlin RL, Kontoyiannis DP, Liu YJ, Arold ST, Gilliet M. 2015. T_H_17 cells promote microbial killing and innate immune sensing of DNA via interleukin 26. Nat Immunol 16:970–979. doi:10.1038/ni.3211. 26168081 PMC4776746

[51] Scott MG, Davidson DJ, Gold MR, Bowdish D, Hancock RE. 2002. The human antimicrobial peptide LL-37 is a multifunctional modulator of innate immune responses. J Immunol 169:3883–3891. doi:10.4049/jimmunol.169.7.3883. 12244186

[52] Liu C, Zheng H, Yang M, Xu Z, Wang X, Wei L, Tang B, Liu F, Zhang Y, Ding Y, Tang X, Wu B, Johnson TJ, Chen H, Tan C. 2015. Genome analysis and *in vivo* virulence of porcine extraintestinal pathogenic *Escherichia coli* strain PCN033. BMC Genom 16:717. doi:10.1186/s12864-015-1890-9. PMC4578781 26391348

[53] Liu C, Chen Z, Tan C, Liu W, Xu Z, Zhou R, Chen H. 2012. Immunogenic characterization of outer membrane porins OmpC and OmpF of porcine extraintestinal pathogenic *Escherichia coli*. FEMS Microbiol Lett 337:104–111. doi:10.1111/1574-6968.12013. 23003111

[54] Howe K, Karsi A, Germon P, Wills RW, Lawrence ML, Bailey RH. 2010. Development of stable reporter system cloning luxCDABE genes into chromosome of *Salmonella enterica* serotypes using Tn7 transposon. BMC Microbiol 10:197. doi:10.1186/1471-2180-10-197. 20653968 PMC2918591

[55] Keller S, Vargas C, Zhao H, Piszczek G, Brautigam CA, Schuck P. 2012. High-precision isothermal titration calorimetry with automated peak-shape analysis. Anal Chem 84:5066–5073. doi:10.1021/ac3007522. 22530732 PMC3389189

[56] Chih YH, Lin YS, Yip BS, Wei HJ, Chu HL, Yu HY, Cheng HT, Chou YT, Cheng JW. 2015. Ultrashort antimicrobial peptides with antiendotoxin properties. Antimicrob Agents Chemother 59:5052–5056. doi:10.1128/AAC.00519-15. 26033727 PMC4505259

